# Issues in Measuring and Interpreting Human Appetite (Satiety/Satiation) and Its Contribution to Obesity

**DOI:** 10.1007/s13679-019-00340-6

**Published:** 2019-04-29

**Authors:** Catherine Gibbons, Mark Hopkins, Kristine Beaulieu, Pauline Oustric, John E. Blundell

**Affiliations:** 1 School of Psychology, University of Leeds, 4 Lifton Place, Leeds, LS2 9JT UK; 2 School of Food Science and Nutrition, University of Leeds, Leeds, LS2 9JT UK

**Keywords:** Appetite control, Satiety, Satiation, Energy balance, Obesity

## Abstract

**Purpose of Review:**

The goals of this paper are to report current research practices in investigations of human appetite control and to assess their relationships with emerging theoretical principles. Appetite is often distinguished by the separation of homeostatic and hedonic processes.

**Recent Findings:**

This report assesses the validity of a homeostatic toolkit to measure subjectively perceived hunger and its relationship to the developing processes of satiation (control of meal size) and satiety (control of the post-eating period). The capacity of a procedure to measure the influence of hedonic processes on food intake is also evaluated. A major issue is the relationship between the pattern of eating behaviour (influenced by the underlying drive to eat and the inhibition induced by the act of eating itself) and the parallel underlying profile of hormonal and other metabolic biomarkers.

**Summary:**

Increasing recognition is being given to individual variability in the expression of appetite, and the fact that the use of the average (mean) response conceals important information about the nature of appetite control. There is a growing interest in the identification of satiety phenotypes that operate in parallel to metabolic phenotypes. Interestingly, energy expenditure (metabolic and behavioural) contributes to an energy balance framework for understanding energy intake (appetite).

## Introduction

The human appetite system is intimately linked to body composition and therefore to obesity. Appetite, by definition, is the system that influences energy intake (food consumption) and associated motivational states such as hunger. Appetite also interacts with, and is influenced by, energy expenditure. Therefore, appetite can be most completely understood in relation to energy expenditure (metabolic and behavioural) and is best considered within an energy balance framework.

It is of enormous significance that humans are omnivores, and this feature confers the capacity to consume a huge range of food materials. This attribute has been critical in allowing humans to evolve and to colonise every part of the planet. The omnivorous habit gives humans the adaptive capacity of food choice. This ability is also relevant in the light of the technological capacity of the modern food industry to produce a massive range of processed foods with a strong sensory appeal. This abundance, and the opportunity for almost unlimited food choices, makes omnivores vulnerable to overconsumption and obesity. In a broad consideration of appetite control, three issues can be distinguished which concern the following: the origins of the drive to eat (hunger), food choice and decisions about what to eat, and the control over the inhibition of eating and the amount of food consumed (how much is eaten). In turn, obesity may arise from a strong drive to eat, inappropriate food choices (for example, for foods with strong sensory appeal and high energy density), or weak inhibition of eating. People vary markedly in the strength and direction of these processes.

It is therefore important that researchers and clinicians have access to procedures for the reliable and valid measurement of the drive to eat (mainly hunger and associated sensations), inhibitory processes over eating (satiation and satiety), and mechanisms that lead to different food choices (for example, the hedonic attributes of liking and wanting). This paper will present a current evaluation of the optimal methods for assessing these processes. In order to interpret the operation of the appetite system, these measurements should be considered in relation to the measurement of total energy expenditure, both metabolic (e.g. resting metabolic rate (RMR)) and behavioural (e.g. physical activity and sedentariness). Within this framework, the variability between individuals becomes apparent, and a way to manage this is through the identification and measurement of phenotypic sub-types (e.g. the high satiety phenotype).

Appetite forms a bridge between the internal and external environments and therefore has both biological and behavioural/psychological aspects. Measurement therefore has to embrace both physiological and behavioural end-points. In turn, the interpretation of appetite requires recognition of both energy intake and energy expenditure, and physiology and behaviour.

## Measuring Satiety

The term satiety is commonly used in the study of biopsychological appetite control. It describes the period between meals (after food consumption) and the many processes occurring at this time. The satiety cascade [1] identifies a number of processes that occur after the consumption of food; therefore, it is important in satiety studies to minimise the number of variables changing simultaneously and, ideally, to ensure only one variable differs between the active and control conditions [2]. Pilot testing should be used to keep active and control conditions as closely matched as possible so that participants cannot detect differences. The timing, nature, and structure of the subsequent test meal itself are crucial to the outcomes of the study. If the test product is a meal, the interval to the next meal should be substantial and reflect normal eating patterns, likewise, if the preload is a snack, the period should be in line with the proposed action of the product. Furthermore, varying the palatability of the test meals will affect the compensatory response to preloads that vary in size [3] and having one large meal compared to a buffet style meal will also affect the results [4]. Due to the large inter-individual variability (discussed more later) in eating behaviour and perceptions of subjective appetite, these studies are optimally performed with repeated measures designs whereby each participant acts as their own control.

Clearly, the optimal study design for measuring human appetite differs across studies; therefore, comparing studies measuring satiety can be difficult. Satiety is generally measured in both the fasting and postprandial period using subjective ratings of hunger/fullness and/or gut peptides such as ghrelin/GLP-1/PYY. These will now be discussed in turn.

### Appetite Rating Scales

When attempting to measure satiety, a consideration of what measure or tool to use is required. Traditionally, satiety is measured through subjective ratings of hunger and fullness. These subjective appetite ratings (a measure of the motivation to eat) are measured through visual analogue scales (VAS) and have been used in clinical and research settings to continuously monitor a range of subjective sensations such as depression, pain, and appetite [5]. These measures provide valuable information on sensations that are difficult to monitor using alternative methods [6]. VAS typically take the form of 100-mm horizontal lines anchored at both ends by extreme subjective feelings [7]. This horizontal line represents a continuum and allows the participant to place a mark on the scale reflecting the intensity of a subjective sensation at a particular time (i.e. state), allowing the sensation to be measured and quantified. The interpretation of VAS is usually unambiguous since the descriptive terms are already present at the end of each line [5]. In some instances, 5-point Likert scales are used; however, preference should be given to 100-mm lines. VAS can be used to ask a variety of questions regarding appetite and often include four basic terms: hunger, fullness, prospective food consumption, and desire to eat (originally devised and validated by Rogers and Blundell [1]). Traditionally, VAS were administered using pen and paper (P&P), which was quick and relatively easy to use. However, data collection from the P&P method is often time-consuming since each line needs to be measured manually and inputted into a spreadsheet individually, a procedure which introduces the possibility of human error. To eliminate the problems of using P&P, portable handheld computers have been developed to administer appetite scales electronically (Electronic Appetite Ratings System or EARS) [8]. The transition to the use of handheld computers was enabled by their relatively inexpensive cost and their associated practical benefits [9]. A number of electronic devices have now been validated for administering VAS with more recent versions allowing participants to use a ‘stylus’ to mark their responses on the screen of the device—which is ergonomically similar to placing a mark on a paper VAS using a pencil [9–12]. A number of studies have used VAS to measure appetite and have shown a high degree of reproducibility [8–11], with a number of reviews supporting their validity and reliability [5, 13, 14]. A range of key experimental studies have utilised the laboratory test meal procedure [15] to confirm the validity and reliability of VAS as a measure of the strength of the motivation to eat [16].

In recent years, the use of subjective VAS has progressed and whilst some research groups use a single component rating (hunger/fullness), others use a composite score of several components to calculate an overall appetite score [17]. This is often calculated through the equation: satiety + fullness + (100 − hunger) + (100 − prospective food consumption)/4. This approach also allows the satiety quotient relative to the energy/weight content of the food provided to be calculated [18–20], allowing assessment of subjective appetite relative to amount of energy consumed. The use of this approach has allowed the identification of distinctive satiety phenotypes. Low satiety phenotypes have recently been shown to be characterised by behavioural (greater energy intake) and psychological (higher cravings, hunger, desire to eat, and prospective consumption) characteristics that are associated with a risk for overconsumption. This is an interesting recent development that requires further investigation, particularly in populations with obesity. In the past, few differences have been identified in terms of appetite sensations when individuals with obesity have been compared to individuals with healthy weight. Their subjective appetite shows similar sensitivity to macronutrients with a rapid decrease in hunger after eating and a steady return to normal after approximately 3 h [21]. Lean and obese groups have also been shown to respond similarly to dietary manipulations; both groups have been shown to reduce intake in response to low- versus high-energy dense foods, but not reduce intake after high- versus low-fat content [22].

Recent work has attempted to investigate the relative importance of individual appetite sensations and their association with energy intake essentially considering the question of whether separate appetite ratings (hunger/fullness) measure the same thing? There is some discrepancy in findings with some research identifying hunger as the single rating that best represented other subjective ratings [23], whilst others have shown fullness [24] and desire to eat/prospective consumption are more closely associated with energy intake [25]. These differences may be due to timing of measurement or technique used which greatly influence the interpretation of findings as previously discussed. However, subjective sensations do not provide the full picture of appetite control and energy intake and other variables are contributing to satiety and satiation.

### Biomarkers of Satiety

Alongside subjective ratings of appetite, there is a need to investigate the strength of satiety through circulating levels of appetite-related peptides. Ghrelin, cholecystokinin (CCK), glucagon-like peptide 1 (GLP-1), and peptide YY (PYY), amongst others, are thought to play a role in the episodic control of appetite and are known to fluctuate around meal times. These peptides are released from several sites throughout the gastrointestinal system. Ghrelin is released from the stomach and is often referred to as the ‘hunger hormone’. It is high during periods of fasting and decreases in response to food intake, therefore being regarded as orexigenic. CCK, GLP-1, and PYY are released from the small and large intestines and are considered satiety peptides. They are low during fasting and increase in response to food consumption and therefore anorexigenic.

When supra-physiological levels of these peptides are infused, it provides evidence for their role in energy intake and appetite control; however, their influence under normal circulating physiological levels is not so profound [26]. Due to the mirrored patterns of hunger, fullness, and appetite-related peptides, they are often measured simultaneously as indicators or biomarkers of satiety. However, it has been noted recently that the evidence for a role of these peptides in short-term appetite is far from clear [26]. The relative importance of individual peptides is unknown and, at present, there is no composite peptide measure similar to a subjective appetite rating. What has become increasingly clear is that peptide levels do differ in obesity, but there is little evidence to suggest peptides are implicated in the cause of obesity, but rather the changes are a consequence of weight gain.

There are several difficulties in the practicality of measuring these peptides. Firstly, the peptides degrade extremely quickly; therefore, consistent procedures need to be put in place to prevent this. Blood samples should be mixed immediately with protease inhibitors with the combination of inhibitors dependent on the range of peptides to be measured. Not surprisingly, these postprandial studies measuring appetite-related peptides are extremely difficult and expensive to carry out. One interesting theoretical and methodological issue is that despite being characterised as a ‘gold standard’ technique, questions remain as to whether or not these peptide biomarkers provide more convincing evidence for satiety than changes in subjective rating scales.

## Measuring Satiation

Satiety is associated with the inter-meal period and does not reflect processes occurring ‘during’ the meal. These processes, known as satiation, bring the meal to an end and therefore determine meal size (energy and/or weight). Obese and healthy weight people have not been shown to differ in the frequency of eating [27], yet people with obesity consume a greater number of calories therefore supporting the importance of meal size as a contributor to over-consumption and obesity. People often refer to fullness and/or changes in perceived taste sensations [28, 29] when asked about factors associated with stopping eating. The quantity and variety of foods provided strongly influence meal size/satiation. Single foods are more likely to elicit stronger sensory responses as the provision of several foods may divert the focus to other sensory components and delay satiation. Researchers should be aware of how decisions around study design can influence the responses of subjects to food provided and therefore the interpretation of underlying processes. The palatability of foods provided to the participants should be verified during the screening process of the study to ensure equi-palatable (to all participants) foods are used to ensure this does not unduly influence measures of satiation.

Environmental/contextual factors that may be involved in meal termination should also be considered [30]. People tend to consume most (if not all) of the food on their plate, even if the foods are not particularly liked. Cognitive factors involved in meal termination imply that over thousands of eating episodes, we ‘learn’ about the satiating effects of food and can therefore estimate the amount needed of each food/meal to elicit satiation. Energy density is a variable of considerable importance when providing meals to measure satiation. On visual inspection, low-energy dense (vegetables, fruit, etc.) foods tend to be larger portion sizes than high dense foods (chocolate, cheese, etc.). This feature of energy density is now well documented [31] and studies measuring satiation should account for this dependent on their research question and design. Texture of food is also an important influence on satiation since liquid foods are consumed at a faster rate than solid foods [32] and consequently more calories are likely to be consumed. When measuring energy intake, it is important that the participants are in a similar state of appetite, since hunger is a clear determinant of food intake. Participants should be limited in their food and drink intake (essentially allowed water only) for a number of hours before being provided with an ad libitum meal to ensure a similar level of hunger between participants and between conditions.

Clearly, a number of parameters can influence satiation, and for a true test of meal termination, only one factor should be allowed to vary at a time. Study designs can be varied significantly, and therefore, it is somewhat questionable how comparable studies of this nature can be. Our viewpoint would be that if the study is designed with consideration of the components mentioned above, then comparisons can be made, but caution should be applied when methodologies differ substantially.

## Measuring Food Hedonics

Whilst not being a direct measure of the amount of food eaten, food reward (i.e. liking and wanting) is still considered under the umbrella of appetite control, as it contributes to determining food choice and consumption. Liking is defined as the pleasure of eating a food and wanting is the drive to eat triggered by a food cue. A recent review investigating the role of food reward in weight management made considerable note of methodological limitations present in the current literature. A key issue was the definition and measurement of food reward as reward processes such as liking and wanting cannot be directly observed [33••]. Successful measures of food reward must reflect the existence of the distinction between liking and wanting. Explicit measures of food ‘liking’ and ‘wanting’ most commonly use psychometric techniques such as numerical scales and VAS. Questions such as ‘How pleasant would it be to taste some of this food now?’ and ‘How much do you want this food?’ are often used for the assessment of explicit liking and wanting. These methods are subject to similar issues as VAS for hunger/fullness such as self-report issues and social desirability. However, they can be effective in deciphering subtle manipulations (i.e. fasting or fed state) and they often predict eating behaviour. Generally, people tend to be proficient in reporting explicit liking, but find it more difficult to determine their implicit wanting for food. Implicit wanting encompasses the motivational aspects of reward-seeking behaviour. ‘Wanting’ measures should be as spontaneous as possible as the measured behaviours are more likely to reflect the core process of ‘wanting’ without contamination from subjective processes. In recent years, a number of methodologies have been put forward to assess more implicit forms of wanting. These techniques tend to include tasks that require a physical response such as a lever press or mouse click in relation to the simulated or actual presence of food or food cues where effort or reaction time is measured. Techniques tend to fall into one of two categories; the first assesses wanting as the reinforcing value of the food or how hard an individual is willing to work to gain access to food compared to an alternative reward [34]. The second technique such as the Stroop task, the Visual Probe task, Stimulus-Response compatibility task, and the Leeds Food Preference questionnaire measures reaction times following exposure to a food compared to a control or alternative food category [35]. The resulting ‘approach bias’ (i.e. the behavioural choice), affected by the attention grabbing/maintaining properties of the food and reflected in the speed of the response, is interpreted as a measure of motivational value or ‘wanting’ [36, 37]. A recent systematic review on the role of food reward in weight management concluded that liking and wanting for high-energy food decreased during weight management, with different types of interventions (behavioural, pharmacological, and cognitive) being equally effective in reducing food reward [33••]. This review also points to the fact that future research should attempt to be consistent in the definitions and techniques used to measure food reward in order to make comparisons in the future possible.

### Individual Variability and Appetite Control

**Fig. 1:**
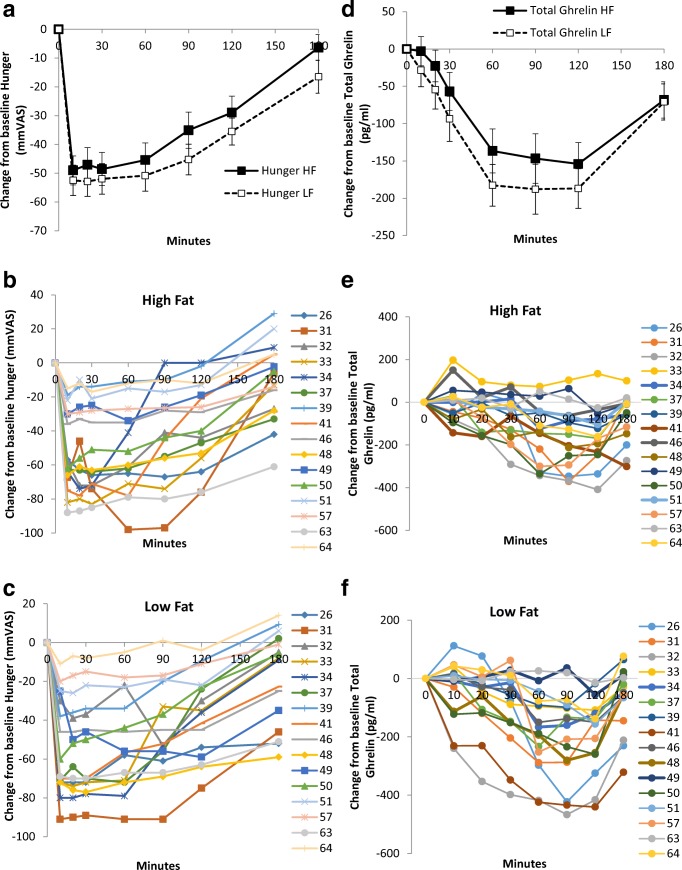
Panel **a** shows the average hunger suppression after high-fat and low-fat meals. Panels **b** and **c** show the individual profiles of hunger for each participant after both high- and low-fat meals. Panel **d** shows the average ghrelin suppression after high-fat and low-fat meals. Panels **e** and **f** show the individual profiles of ghrelin for each participant after both high- and low-fat meals

A salient feature of the appetite-related processes described above is the inherent inter-individual variability typically observed in these processes. Whilst studies typically display the mean pattern of response following the consumption of a fixed energy test meal (or other experimental manipulations such as a bout of exercise), examination of the individual responses will typically reveal large variability in the individual profiles of subjective appetite ratings and peptides such as GLP-1 and ghrelin (see Fig. 1 and [38] for example mean and individual profiles). Such variability also exists in responses to acute and chronic exercise [39–44], with King et al. [45] reporting marked inter-individual variability in hunger, acylated ghrelin, and ad libitum energy intake in response to a single bout of aerobic exercise in young healthy adult males for example. Given the key role that these processes play in the overall expression of food intake, it is not perhaps surprising that this heterogeneity is echoed in the marked inter-individual variability seen in weight loss following lifestyle (diet and exercise) [39–42, 46–48], pharmacological [49, 50], and surgical [51–53] weight loss interventions.

Inter-individual variability in measures such as hunger, satiety, and food ‘liking’ and ‘wanting’ may help account for the diversity seen in eating behaviours between individuals and, in part, help account for differences in the susceptibility/resistance to weight loss or gain. An inability to recognise and respond to internal sensations of hunger [54], or a weakened satiety response to food consumption [55], is thought to be a risk factor for overconsumption and weight gain. The individual variability observed in these processes has prompted some to try and use an individual’s response (i.e. magnitude and/or direction) to an experimental manipulation to identify discordant or dichotomous phenotypes, which help explain differences in the biological or behavioural outcomes of a study. For example, individuals have been categorised based on whether they ate more (i.e. compensators) or less (i.e. non-compensators) in response to acute [44, 56, 57] or chronic exercise [39, 58] to examine for mechanistic differences between groups. Using a similar approach, the baseline postprandial response to meal ingestion (greater suppression of acylated ghrelin and greater release of GLP-1 and total PYY) [59] and changes in food ‘liking’ and ‘wanting’ [60] have been shown to differentiate between those susceptible and resistant to exercise-induced weight loss. It has also been proposed that a low satiety phenotype exists in which some individuals report a weakened satiety response following consumption of a fixed energy test meal [24, 55, 61, 62••], and this blunted satiety response may promote overconsumption and weight gain in these individuals.

Recognition that individuals will not all respond in the same manner to a standardised treatment or manipulation represents an important step in the development of more personalised obesity treatments and may help identify individuals or sub-groups that benefit from an intervention (despite no apparent mean improvement in the intervention group). However, whether such research leads to more efficacious weight loss interventions remains unclear at present, and there is currently a lack of robust biomarkers or predictors of how an individual will respond to lifestyle interventions. There has also been debate over how ‘true’ individual variability can be identified, i.e. variability that is distinct from that induced by measurement error and/or random within-subject variability [63]. This issue was recently addressed by Goltz et al. [64], who examined the reproducibility of the individual responses in subjective appetite, acylated ghrelin, and total PYY following an acute bout of exercise. Using a replicated cross-over design in which 15 active men completed two control and two exercise conditions in a random order, good reproducibility was seen in the exercise-induced changes for subjective appetite ratings and appetite-related peptides. Furthermore, the inter-individual variability in these responses exceeded that which could explained by measurement error and random within-subject variability. As such, these findings suggest the inter-individual variability in appetite-related processes are not just an artefact of measurement or random error, and these findings are in agreement with previous studies examining the reproducibility of within-subject subjective appetite scores [5, 14] and exercise-induced changes in subjective appetite and energy intake [65, 66].

### Energy Expenditure, Body Composition, and Energy Intake—Importance for Obesity

Thus far, we have discussed the measurement of satiety and satiation, which are short-term measures of appetite control. It can be questioned whether these episodic measures are sufficient to understand appetite control without consideration of longer term influences. Energy balance and resulting effects on body weight, and thus obesity, are the product of a complex relationship between energy intake (EI) and energy expenditure (EE). Total daily energy expenditure (TDEE) is composed of resting metabolic rate (RMR), together with physical activity (PA) energy expenditure and thermic effect of food [67]. RMR (largely determined by fat-free mass) has been found to be strongly positively associated with EI in lean individuals and those with overweight and obesity [68–71, 72•] and has led to the suggestion that it exerts a tonic day-to-day signal for hunger and the drive to eat [73]. In order to properly assess RMR, it is important to use objective measurements via indirect calorimetry following standard operating procedures and guidelines. For example, participants should refrain from exercise, caffeine, and alcohol for an extended period prior to the test, be overnight fasted, and the room should be thermo-neutral to achieve optimal results [74].

Alongside RMR, body composition—which includes fat mass and fat-free mass, has been proposed as another tonic signal of appetite. Common laboratory assessment methods are based on two-compartment models of fat mass and fat-free mass which include air displacement plethysmography, dual x-ray absorptiometry, and bioelectrical impedance, with the ‘gold standard’ method being the four-compartment model which takes into account body mass, total body volume, total body water, and bone mineral content to calculate body fat [75]. Body composition should be measured in a fasted and euhydrated state, using standard operating procedures specific to the equipment. The role of fat-free mass as a driver of the motivation to eat has been shown to be fully mediated by its influence on RMR [76]. In contrast, fat mass has been proposed to exert an opposing, inhibitory role on food intake, but the evidence is less consistent [68–71, 77]. However, negative feedback signals reflecting energy stores inhibiting food intake appear to be blunted with higher body fat [78], and most of the studies investigating the role of body composition in appetite control have been conducted in individuals with overweight and obesity. This inhibitory role of fat mass on energy intake may also be mediated by psychological factors as it was recently found that cognitive restraint mediated the relationship between fat mass and energy intake [79••]. Thus, physiological and psychological/cognitive factors are likely to interact in determining food intake.

The contribution of PA, the behavioural component of energy expenditure, towards the drive to eat is less apparent [80, 81]. Compared to RMR, PA makes up a smaller portion of TDEE and is more variable; therefore, its impact on EI may be harder to quantify. However, it has been proposed that habitual PA is a determinant of EI just like RMR (but with great individual variability) [82]. PA encompasses structured exercise in addition to occupational, household, transportation activity, and other activities of daily living, termed non-exercise activity thermogenesis [83]. The proportion of each can vary widely between and within individuals depending on levels of physical activity and daily exercise regime. Additionally, sedentary behaviour and physical inactivity can also influence appetite although it is important to distinguish between them. Sedentary behaviour can be defined as ‘any waking behaviour characterized by an energy expenditure ≤1.5 metabolic equivalents (METs), while in a sitting, reclining or lying posture’, whereas physical inactivity is ‘an insufficient physical activity level to meet present physical activity recommendations’ [84]. In recent years, research-grade wearable technologies have allowed for the estimation of free-living TDEE, and minutes spent sedentary and in different intensities of PA (i.e. light, moderate, and vigorous), which has overcome a major limitation of past research. Technological advancements have also allowed for the measurement of the postural element of sedentary behaviour, and a novel integration technique with accelerometry has produced a ‘true’ objective assessment of sedentary behaviour which incorporates sleep, activity intensity, and postural dimensions [85].

Evidence suggests that PA influences appetite control through a dual-process action which increases the drive to eat but also strengthens post-meal satiety [58]. Indeed, several studies and a systematic review have found that the effects of habitual PA level on EI is characterised by a J-shape relationship [86–88]. At higher levels of PA, daily EE and EI are closely matched. But at lower levels of PA where body mass is also greater, the reduction in EE is not matched by a reduction in EI but an increase, such that daily EI exceeds EE. Physically active individuals show better compensation following consumption of preloads differing in energy content and reduce EI to offset the difference in energy consumed from the preloads, compared with their less active counterparts [89–94]. These improvements in satiety may be associated with long-term exercise-induced adaptations such as episodic satiety signalling [90, 95, 96] or gastric emptying [97]. In addition, evidence shows that habitual PA is associated with reductions in body fat, which may mediate some of the improvements seen with both homeostatic and hedonic appetite control systems [88, 98•]. However, the inter-relationships between PA, EE, body composition, and appetite control remain to be fully understood.

## Conclusion

This report has addressed issues in human appetite control that are relevant for understanding and managing obesity. Appetite reflects the expression of the motivation to eat and the behaviour that is directed towards consumption of food and drink items available in the environment. Nutrition and eating behaviour are inextricably linked since behaviour is the agency through which nutrients enter the body. In most societies, the nutritional environment is replete with a huge range of highly processed foods engineered with strong sensory appeal and backed up by intensive marketing. Many observers believe that the food environment is largely responsible for the current high prevalence of obesity. Therefore, understanding the patterns of behaviour and sensations that mediate the impact of the environment upon body composition are of major importance.

To be of value for understanding obesity, appetite measures should comprise a robust set of measuring instruments (appetite toolkit) that provide a means of evaluating the strength of the motivation to eat, the operation of key food choices, and hedonic processes that modulate the homeostatic system. Appetite is concerned with energy intake, but recent theoretical and practical developments have indicated that energy expenditure (metabolic and behavioural) plays a major role in driving and modulating food intake. Therefore, to construct a complete picture tools used to measure energy intake should be used alongside tools to measure energy expenditure.

Biological variability in human appetite is further emerging and a recently recognised factor relevant to obesity. Individual differences in the profiles of hunger, so-called satiety peptides and food choices, are consistent with the wide variability in the characteristics and habits of people with obesity. This means that there is no single statement about appetite that explains obesity; rather the various appetite toolkits provide a way of describing obesity in different populations and in distinct environments. The concept of appetite phenotypes (such as the ‘low satiety’ phenotype) is comparable to body composition phenotypes [99] and is becoming recognised as a useful way of managing this individual complexity in physiological and behavioural systems.

Currently, measures of human appetite control remain vital for assessing the influence of a range of factors on energy intake including the following: the form and composition of foods (e.g. energy density and portion size), anti-obesity drugs, sensory food quality, and physical activity regimes. In addition, appetite control tools help to establish theoretical principles important in mediating the overall effect of the environment on body composition in people with obesity. The evidence cited here indicates that the portfolio of tools available is capable of disclosing both substantial and subtle effects. Human appetite is an extremely complicated aspect of human functioning, but a scientific approach makes it relevant for the study of obesity.
